# Protective effect and possible mechanisms of resveratrol in animal models of spinal cord injury: a preclinical systematic review and meta-analysis

**DOI:** 10.3389/fimmu.2026.1853441

**Published:** 2026-05-21

**Authors:** Yingchun Li, Dongping Wan, Dongliang Wang, Tao Chen, Rui Wang, Rui Tang, Tianle Chen, Shihang Cao, Bo Dong, Baohui Wang

**Affiliations:** 1 Honghui Hospital, Xi’an Jiaotong University, Xi’an, Shaanxi, China; 2 The First Clinical Medical College, Guangxi University of Chinese Medicine, Nanning, Guangxi Zhuang Autonomous Region, China; 3 Department of Orthopedics and Traumatology, The Affiliated Traditional Chinese Medicine Hospital, Southwest Medical University, Luzhou, Sichuan, China; 4 The First Clinical Medical College, Chengdu University of Traditional Chinese Medicine, Chengdu, Sichuan, China

**Keywords:** animal model, locomotor recovery, meta-analysis, neuroprotection, resveratrol, spinal cord injury

## Abstract

**Background:**

Resveratrol has shown potential neuroprotective effects in experimental spinal cord injury (SCI), but its overall efficacy has not been systematically evaluated. This study aimed to assess the effects of resveratrol on locomotor recovery and related pathological and biochemical outcomes in animal models of SCI.

**Methods:**

A systematic review and meta-analysis was conducted on controlled *in vivo* studies of resveratrol in experimentally induced SCI models, including traumatic and ischemia-reperfusion models. Major locomotor, oxidative stress, inflammatory, apoptotic, and edema-related outcomes were pooled as standardized mean differences (SMDs) with 95% confidence intervals (CIs).

**Results:**

In total, 38 studies were included. Resveratrol significantly improved locomotor recovery, as reflected by higher BBB scores at 3, 7, 14, 21, and 28 days after injury (SMDs ranging from 2.56 to 5.23) and higher BMS scores at the corresponding time points (SMDs ranging from 1.40 to 3.37). It also attenuated oxidative stress, with reduced MDA levels at 24 h, 3 days, and 7 days, and increased SOD and GSH levels. Inflammatory responses were suppressed, as shown by lower MPO, TNF-α, IL-1β, and IL-6 levels, while IL-10 showed a borderline increase at 7 days. In addition, resveratrol reduced the TUNEL-positive rate and spinal cord water content, suggesting a possible reduction in apoptotic cell death and a potential effect on spinal cord edema based on spinal cord water content. However, apoptosis-related molecular markers showed heterogeneous results, with no significant changes in BCL-2 and caspase-3 at some time points and an increased BAX level at 7 days.

**Conclusions:**

Current preclinical evidence supports that resveratrol exerts neuroprotective effects in SCI animal models by improving locomotor recovery and modulating oxidative stress, inflammation, apoptosis-related changes, and edema, although molecular evidence for apoptosis-related pathways remains heterogeneous.

**Systematic Review Registration:**

https://www.crd.york.ac.uk/prospero/, identifier CRD420261289830.

## 1 Introduction

Spinal cord injury (SCI) is a severe injury to the central nervous system that can result in varying degrees of sensory, motor, and autonomic dysfunction (1–3). In 2021, SCI was estimated to account for approximately 574,502 incident cases worldwide, with a prevalence of 15,400,682 cases and 4,566,237 years lived with disability (YLDs) (4). Globally, around 500,000 people are paralyzed each year due to traumatic SCI, underscoring the urgent need for effective therapies to improve post-injury locomotor function and overall quality of life (5). The pathophysiology of SCI is highly complex and generally involves a primary mechanical insult followed by a cascade of secondary injury processes (6). Primary injury results from the direct impact of external force on the spinal cord tissue (7), whereas secondary injury encompasses a series of pathological events, including inflammation, oxidative stress, apoptosis, mitochondrial dysfunction, blood–spinal cord barrier disruption, and glial scar formation, all of which further aggravate neural tissue damage and hinder neurological recovery (8–10). Because secondary injury evolves within a definable therapeutic time window, pharmacological interventions targeting this stage are considered a major research focus for improving SCI outcomes.

Despite continuous advances in basic and clinical research in recent years, the clinical benefits achieved with current approaches remain limited. Unfortunately, no effective strategy is currently available to repair SCI, and affected individuals often suffer from long-term functional impairment and lifelong disability (11). At present, commonly used treatments for SCI include early decompression, surgical stabilization, rehabilitation training, and certain pharmacological interventions; however, their overall efficacy remains unsatisfactory (12, 13). Therefore, the search for novel candidate agents with multitarget neuroprotective effects has remained a major focus in SCI research. Natural bioactive compounds have attracted increasing attention because of their wide availability, diverse pharmacological activities, and potential safety advantages.

Resveratrol (3,5,4′-trihydroxystilbene) is a naturally occurring polyphenolic micronutrient found in approximately 70 plant species, including grapes, berries, peanuts, and pine trees (14, 15). Previous studies have shown that resveratrol exerts a wide range of biological effects, including antioxidant, anti-inflammatory, and anti-apoptotic activities, as well as modulation of mitochondrial function, improvement of microcirculation, and promotion of autophagy (16–19). In studies of neurological disorders, resveratrol has been demonstrated to confer protective effects in cerebral ischemia, Alzheimer’s disease, Parkinson’s disease, and traumatic neural injury (20–23). In recent years, an increasing number of animal studies have suggested that resveratrol may exert neuroprotective effects in SCI by suppressing the release of inflammatory mediators, attenuating oxidative stress, regulating cellular energy metabolism, promoting neuronal survival, and enhancing locomotor recovery (24–27). However, substantial heterogeneity exists across studies in terms of animal species, injury models, dosage regimens, routes of administration, treatment duration, and outcome measures, making the overall effect and robustness of the current evidence remain uncertain.

Systematic reviews and meta-analyses can synthesize preclinical evidence, evaluate the overall effects of interventions, and explore potential sources of heterogeneity. Although resveratrol has shown potential neuroprotective effects in animal models of SCI, its overall efficacy has not been systematically evaluated. Therefore, this study conducted a systematic review and meta-analysis to assess the effects of resveratrol on locomotor recovery and related pathological and biochemical outcomes in SCI animal models.

## 2 Methods

This study was registered in PROSPERO (CRD420261289830).

### 2.1 Literature search

We systematically searched PubMed, EMBASE, Web of Science, Scopus, CNKI, Wanfang, Sinomed, and VIP databases from inception to December 2025 to identify preclinical animal studies investigating the effects of resveratrol on spinal cord injury (SCI). Search strategies were tailored to each database using combinations of controlled vocabulary and free-text terms related to “resveratrol,” “spinal cord injury,” “SCI,” “animal model,” and “preclinical study.” In addition, the reference lists of relevant articles and reviews were manually screened to identify potentially eligible studies missed by the electronic search.

### 2.2 Inclusion and exclusion criteria

We included controlled *in vivo* animal studies that met the following criteria: (1) SCI was experimentally induced, including traumatic models such as contusion, compression, transection, hemisection, and clip-compression models, as well as spinal cord ischemia-reperfusion injury models; (2) resveratrol was administered as the primary intervention, with a clearly reported dose, route, and timing; (3) the comparator group received vehicle, saline, or no treatment; and (4) at least one predefined outcome of interest was reported.

Eligible outcomes included locomotor recovery assessed by the Basso, Beattie, and Bresnahan (BBB) score or other validated behavioral scales, spinal cord edema, inflammatory cytokines (e.g., TNF-α, IL-1β, and IL-6), oxidative stress markers (e.g., MDA and SOD), apoptosis-related indices (e.g., caspase-3, Bcl-2, Bax, TUNEL-positive cells), histopathological changes, and other biochemical or molecular outcomes relevant to secondary SCI.

We excluded reviews, editorials, conference abstracts, case reports, clinical studies, *in vitro* experiments, non-experimentally induced SCI models, non-rodent studies when data were not comparable, and studies lacking sufficient information for data extraction. Studies were also excluded if resveratrol was used in combination with other interventions and its independent effect could not be determined, or if key treatment details such as dose or administration timing were not clearly described. Titles and abstracts were screened first, followed by full-text assessment for potentially eligible records.

### 2.3 Data extraction

Two reviewers independently extracted data using a predefined and pilot-tested form, and disagreements were resolved through discussion and consensus. For each included study, we collected the following information: publication details (first author and year), animal characteristics (species, strain, sex, age, body weight, and sample size), SCI model characteristics (injury type, spinal segment, modeling procedure, and anesthetic information where available), and intervention details (resveratrol dose, route of administration, treatment initiation time, frequency, duration, and control condition).

Outcome data were extracted for all eligible endpoints at the reported time points. When multiple resveratrol dose groups were included in a single study, data from each eligible comparison were extracted separately; where necessary, shared control groups were handled according to standard meta-analytic methods to avoid double counting. For studies presenting results only in graphical form, numerical data were obtained by contacting the corresponding authors when possible or by digitizing the figures using appropriate software.

### 2.4 Quality assessment

Methodological quality and risk of bias were independently assessed by two reviewers using the SYRCLE risk-of-bias tool for animal studies. The following domains were evaluated: sequence generation, baseline characteristics, allocation concealment, random housing, blinding of caregivers and investigators, random outcome assessment, blinding of outcome assessment, incomplete outcome data, selective outcome reporting, and other potential sources of bias.

Each domain was judged as “low risk,” “high risk,” or “unclear risk” based on the information reported in the original article. Disagreements between reviewers were resolved by discussion after rechecking the full text until consensus was reached.

### 2.5 Statistical analysis

Continuous data were synthesized using RevMan 5.3 and Stata 16.0. Because different studies may have used different measurement scales or units, pooled effect sizes were calculated as standardized mean differences (SMDs) with 95% confidence intervals (CIs). A two-sided p value < 0.05 was considered statistically significant.

Statistical heterogeneity among studies was assessed using the I² statistic. When heterogeneity was low (I² < 50%), a fixed-effect model was applied; otherwise, a random-effects model was used. Prespecified subgroup analyses were performed, where data permitted, according to factors such as animal species, injury model, resveratrol dose, route of administration, treatment timing, and treatment duration. Sensitivity analyses were conducted by excluding studies one by one to evaluate the robustness of the pooled estimates. Publication bias or small-study effects were assessed using Egger’s test when a sufficient number of studies were available. In addition to quantitative synthesis, mechanistic findings related to the neuroprotective effects of resveratrol in SCI were summarized narratively.

## 3 Results

### 3.1 Search results

**Figure 1:**
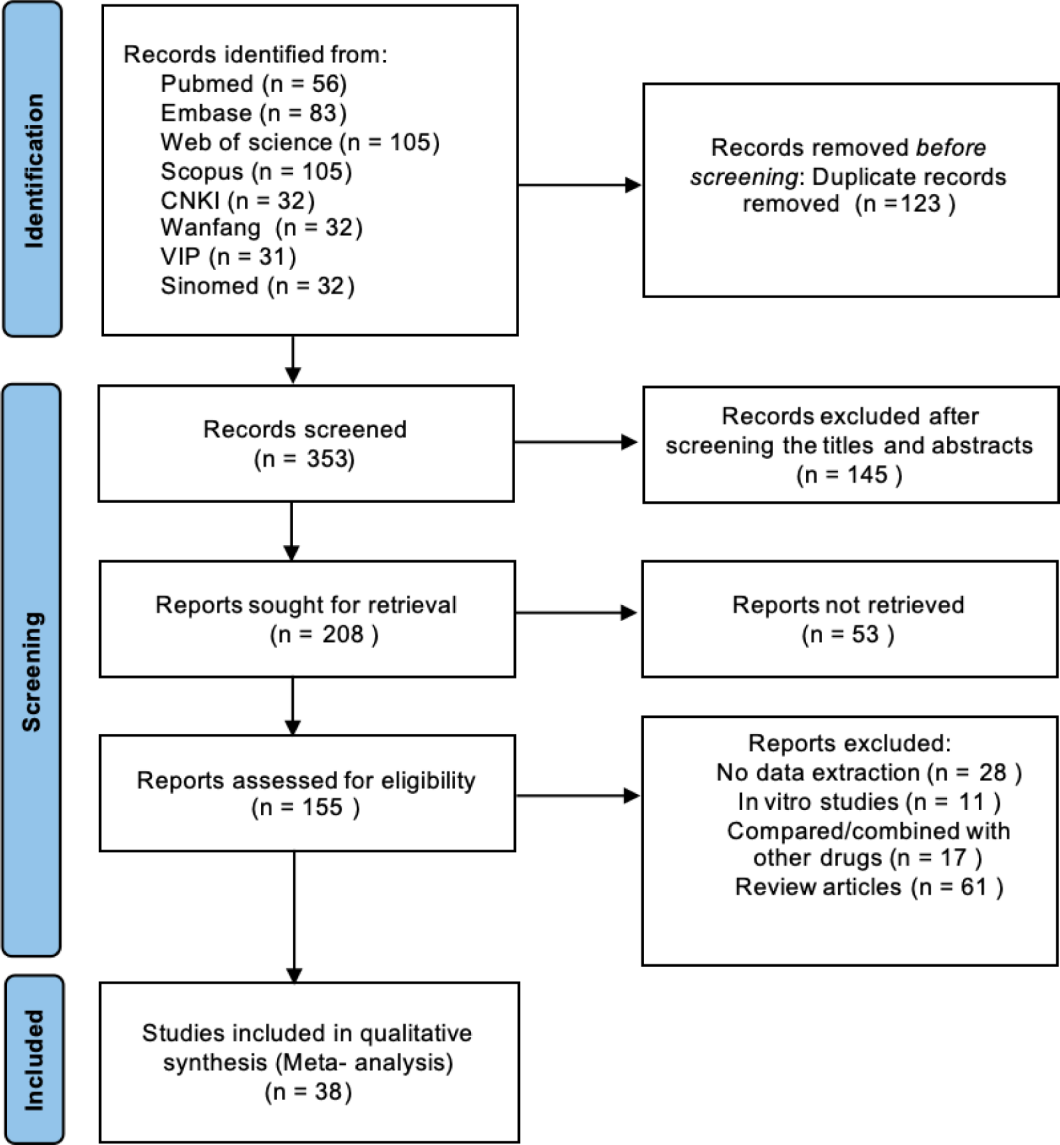
PRISMA flow diagram of study selection.

A total of 476 records were identified through database searching, including PubMed (n = 56), Embase (n = 83), Web of Science (n = 105), Scopus (n = 105), CNKI (n = 32), Wanfang (n = 32), VIP (n = 31), and SinoMed (n = 32). After removal of 123 duplicate records, 353 records remained for title and abstract screening, of which 145 were excluded. A total of 208 reports were sought for retrieval, and 53 could not be retrieved. The remaining 155 reports were assessed for eligibility, and 117 were excluded for the following reasons: no extractable data (n = 28), *in vitro* studies (n = 11), comparison or combination with other drugs (n = 17), and review articles (n = 61). Ultimately, 38 studies were included in the qualitative synthesis and meta-analysis (**Figure 1**).

### 3.2 Characteristics of the included studies

This meta-analysis included 38 (26–63) animal studies published between 2002 and 2025. Most studies used Sprague–Dawley rats (n = 26), and Allen’s weight-drop model was the most common SCI model (n = 30). The injury was mainly induced at thoracic levels, especially T8–T10. Intraperitoneal injection was the predominant route of administration (n = 25), and study duration ranged from 24 hours to 56 days. Detailed characteristics of the included studies are summarized in **Table 1**.

**Table 1:** . Characteristics of the included studies.

First author	Year	Species	Gender	Induction of SCI	Spinal Cord	Methods of administration	Intervention		Sample size		Duration of study
							IG	CG	IG	CG	
Yang	2002	SD rats	Male and female (1:1)	Allen’s weight-drop model	T8	Intraperitoneal injection	100mg/kg	DMSO	6	6	48h
Yang Y	2002	SD rats	Both sexes	Allen’s weight-drop model	T8	Intraperitoneal injection	100mg/kg	DMSO	6	6	48h
Yang	2003	SD rats	Both sexes	Allen’s weight-drop model	T8	Intraperitoneal injection	100mg/kg	DMSO	6	6	48h
Sadi Kaplan	2005	New Zealand White Rabbit	Male	Abdominal aortic occlusion	L4-L5	Intravenous injection	0.1mg/kg	Normal saline	8	8	24h
Liu	2005	SD rats	Male	Allen’s weight-drop model	T8	Intraperitoneal injection	100mg/kg	DMSO	6	6	72h
Ozkan ATES	2006	Wistar rats	Male	Allen’s weight-drop model	T7-T10	Intraperitoneal injection	100mg/kg	NR	6	6	6w
Liu	2009	SD rats	Both sexes	Allen’s weight-drop model	T8	Intraperitoneal injection	100mg/kg	Normal saline	8	8	72h
Liu CJ	2009	SD rats	Both sexes	Allen’s weight-drop model	T8	Intraperitoneal injection	200mg/kg	Normal saline	8	8	72h
Zhao	2010	SD rats	Both sexes	Allen’s weight-drop model	T8	Intraperitoneal injection	100mg/kg	Normal saline	6	6	168h
Liu	2010	SD rats	NR	Allen’s weight-drop model	T8	Intraperitoneal injection	200mg/kg	Normal saline	12	12	72h
Mei	2011	SD rats	Male	Allen’s weight-drop model	T8	Oral gavage	100mg/kg	NR	6	6	72h
Mei	2012	SD rats	Male	Allen’s weight-drop model	T8	Oral gavage	100mg/kg	NR	6	6	7d
Liu	2013	SD rats	Both sexes	Allen’s weight-drop model	T8	Intraperitoneal injection	200mg/kg	Normal saline	3	3	72h
Liu C	2013	SD rats	Both sexes	Allen’s weight-drop model	T8	Intraperitoneal injection	200 mg/kg	Normal saline	3	3	72h
Liu CJ	2013	SD rats	Both sexes	Allen’s weight-drop model	T8	Intraperitoneal injection	200 mg/kg	Normal saline	6	6	72h
Mei	2013	SD rats	Male	Allen’s weight-drop model	T8	Oral gavage	100mg/kg	NR	6	6	7d
Liu	2014	SD rats	Both sexes	Allen’s weight-drop model	T8	Intraperitoneal injection	200mg/kg	Normal saline	10	10	72h
Liu Hui	2014	Wistar rats	Male and female (1:1)	Spinal cord ischemia–reperfusion injury model	L3-L5	NR	40mg/kg	Normal saline	12	12	7d
Mei	2014	SD rats	Male	Allen’s weight-drop model	T8	Oral gavage	100mg/kg	NR	6	6	7d
Zhao	2017	SD rats	Female	Allen’s weight-drop model	T9-T10	Intraperitoneal injection	100mg/kg	DMSO	5	5	28d
Meng	2018	SD rats	Male	Allen’s weight-drop model	T9-T10	Intraperitoneal injection	100mg/kg	DMSO	5	5	21d
Wang	2018	SD rats	Female	Allen’s weight-drop model	T10	Intraperitoneal injection	200 mg/kg	Normal saline	5	5	28d
Liu	2018	SD rats	Male	Allen’s weight-drop model	T9-T10	Intraperitoneal injection	100 mg/kg	Normal saline	36	18	35d
Wang XW	2018	SD rats	Female	Dural incision with 2-mm spinal cord excision	T9	Intraperitoneal injection	100 mg/kg	NR	10	10	8d
Jiang	2020	Japanese big eared rabbit	Male	Allen’s weight-drop model	T10	Intravenous injection	100mg/kg/	Normal saline	10	10	14d
Zhao	2021	SD rats	Male	Allen’s weight-drop model	T9-T10	Intraperitoneal injection	30 mg/kg	Distilled water	5	5	28d
Cheng	2021	C57BL/6 mice	Male	Allen’s weight-drop model	T9-T10	Intraperitoneal injection	200 mg/kg	Normal saline	5	5	3d
He	2022	C57BL/6 mice	Female	Allen’s weight-drop model	T10	Oral gavage	200 mg/kg	PBS	12	12	35d
Jiang	2022	C57BL/6 mice	Male and female (1:1)	Allen’s weight-drop model	T9	Intravenous injection	5 mg/kg	NR	6	6	28d
Li	2022	SD rats	Male	Spinal cord compression model	T8	Intraperitoneal injection	40 mg/kg	Normal saline	10	10	14d
Li Yq	2022	C57BL/6 mice	Male and female (1:1)	Allen’s weight-drop model	T9-T10	Intravenous injection	5 mg/kg	Normal saline	6	6	28d
Han	2023	SD rats	Male	Allen’s weight-drop model	T10	Intrathecal injection	300 μg/10 μl	NR	8	8	21d
Kan	2023	Wistar rats	Female	MASCIS Impactor Model III	T10	Intraperitoneal injection	10 mg/kg	Normal saline	16	16	56d
Ni	2023	C57BL/6 mice	Female	Spinal cord contusion model	T10	Intraperitoneal injection	200 mg/kg	NR	8	8	35d
Esra Aslan	2021	Wistar rats	Female	Spinal cord ischemia–reperfusion injury model	NR	NR	50 mg/kg	NR	6	6	8d
Liu Xb	2024	C57BL/6 mice	Male and female (1:1)	Allen’s weight-drop model	T9-T10	Intravenous injection	5 mg/kg	Normal saline	3	3	28d
Li	2024	SD rats	NR	Allen’s weight-drop model	T12	Intraperitoneal injection	100mg/kg	Normal saline	6	6	14d
Feng	2025	SD rats	Female	Spinal cord injury induced by impactor	T10	Intraperitoneal injection	100mg/kg	DMSO	6	6	7d

### 3.3 Quality assessment of the included studies

**Figure 2:**
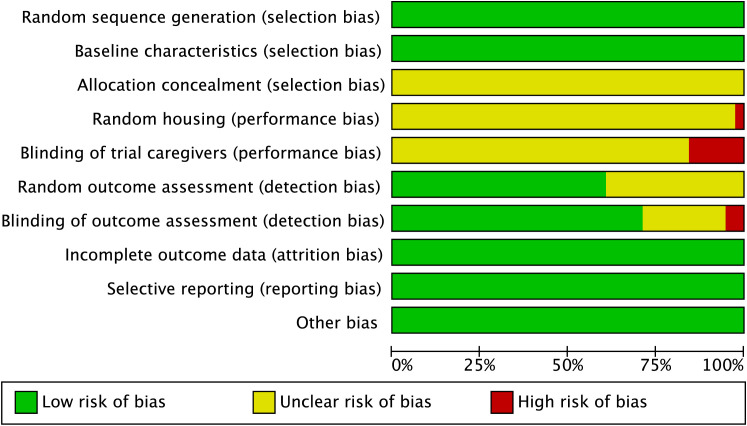
Risk-of-bias assessment of the included studies.

The risk-of-bias assessment indicated that most studies showed low risk in several domains, particularly baseline characteristics, incomplete outcome data, selective reporting, and other bias. However, unclear risk was common for allocation concealment, random housing, and random outcome assessment, largely because of poor reporting. Blinding-related domains also showed unclear or high risk in some studies. Overall, the included studies were of moderate methodological quality. A detailed assessment of study quality is provided in **Figure 2**.

### 3.4 BBB and subgroup analysis

**Figure 3:**
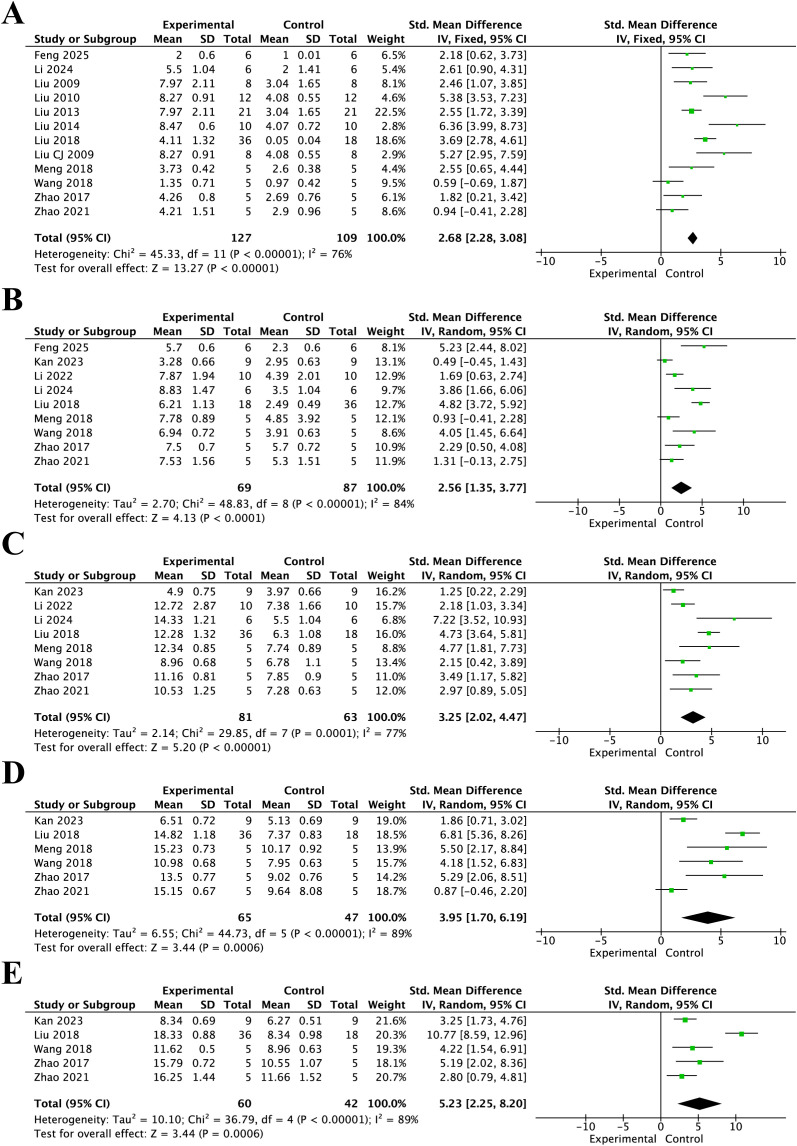
Forest plots of BBB scores showing the effects of resveratrol on locomotor recovery at different time points after spinal cord injury. **(A)** 3 days after injury; **(B)** 7 days after injury; **(C)** 14 days after injury; **(D)** 21 days after injury; and **(E)** 28 days after injury.

As shown in **Figures 3A–E**, BBB scores in the resveratrol treatment group were significantly higher than those in the control group at different post-injury time points. At 3 days after injury (**Figure 3A**), the pooled analysis showed a significant increase in BBB scores in the resveratrol group compared with the control group (SMD = 2.68, 95% CI = 2.28 to 3.08, p < 0.01). At 7 days (**Figure 3B**), BBB scores remained significantly improved in the resveratrol group (SMD = 2.56, 95% CI = 1.35 to 3.77, p < 0.01). At 14 days (**Figure 3C**), the pooled effect size was SMD = 3.25 (95% CI = 2.02 to 4.47, p < 0.01). At 21 days (**Figure 3D**), resveratrol also significantly increased BBB scores compared with the control group (SMD = 3.95, 95% CI = 1.70 to 6.19, p < 0.01). At 28 days (**Figure 3E**), the beneficial effect remained significant (SMD = 5.23, 95% CI = 2.25 to 8.20, p < 0.001).

Subgroup analyses of BBB scores were conducted at 3, 7, 14, 21, and 28 days after SCI according to dose category, timing of treatment initiation, and treatment regimen. At 3 days after SCI (**Supplementary Table 1**), significant improvements in BBB scores were observed across all analyzed subgroups. In the dose-category analysis, the pooled effect sizes were 1.69 (95% CI = 0.19 to 3.18) for low dose (≤50 mg/kg), 2.76 (95% CI = 1.98 to 3.54) for medium dose (100 mg/kg), and 3.85 (95% CI = 1.88 to 5.81) for high dose (≥200 mg/kg), suggesting a trend toward greater benefit with increasing dose. For treatment initiation, both immediate initiation (SMD = 2.85, 95% CI = 1.93 to 3.78) and delayed initiation (SMD = 2.91, 95% CI = 0.39 to 5.44) were associated with significant improvement. Similarly, beneficial effects were found across treatment regimens, including single-dose administration (SMD = 2.82, 95% CI = 1.58 to 4.06), 3-day administration (SMD = 3.54, 95% CI = 1.35 to 5.73), and ≥7-day administration (SMD = 2.40, 95% CI = 1.09 to 3.72). At 7 days (**Supplementary Table 2**), all subgroup analyses also showed significant beneficial effects. The pooled effect size was smaller in the <100 mg/kg subgroup (SMD = 1.11, 95% CI = 0.33 to 1.88) than in the ≥100 mg/kg subgroup (SMD = 3.42, 95% CI = 1.85 to 4.99). Significant improvements were likewise observed in both the immediate initiation subgroup (SMD = 2.82, 95% CI = 0.99 to 4.66) and the delayed initiation subgroup (SMD = 2.01, 95% CI = 0.82 to 3.20). With respect to treatment regimen, both single-dose administration (SMD = 1.56, 95% CI = 0.81 to 2.31) and repeated administration (SMD = 3.16, 95% CI = 1.26 to 5.06) were associated with higher BBB scores than controls. At 14 days (**Supplementary Table 2**), consistent beneficial effects were again observed across all subgroups. In the dose analysis, the pooled effect estimates were 1.88 (95% CI = 1.01 to 2.75) for <100 mg/kg and 4.14 (95% CI = 2.71 to 5.57) for ≥100 mg/kg. Immediate initiation (SMD = 3.16, 95% CI = 1.45 to 4.88) and delayed initiation (SMD = 3.51, 95% CI = 1.29 to 5.73) both remained significant. Likewise, both single-dose administration (SMD = 3.00, 95% CI = 1.57 to 4.43) and repeated administration (SMD = 3.34, 95% CI = 1.49 to 5.19) showed beneficial effects. At 21 days (**Supplementary Table 2**), significant improvement in BBB scores was still observed in most subgroups. In the dose-category analysis, both <100 mg/kg (SMD = 1.42, 95% CI = 0.46 to 2.39) and ≥100 mg/kg (SMD = 5.93, 95% CI = 4.72 to 7.15) subgroups showed significant effects, with a markedly larger pooled estimate in the higher-dose subgroup. In terms of treatment initiation, the immediate initiation subgroup remained significant (SMD = 4.65, 95% CI = 2.19 to 7.12), whereas the delayed initiation subgroup did not show a statistically significant effect (SMD = 0.87, 95% CI = -0.46 to 2.20). For treatment regimen, both single-dose administration (SMD = 5.39, 95% CI = 3.07 to 7.71) and repeated administration (SMD = 3.38, 95% CI = 0.60 to 6.17) remained beneficial. At 28 days (**Supplementary Table 3**), the favorable effect of resveratrol persisted in all analyzed subgroups. The pooled effect sizes were 3.09 (95% CI = 1.88 to 4.30) for <100 mg/kg and 6.81 (95% CI = 2.42 to 11.20) for ≥100 mg/kg. Both immediate initiation (SMD = 5.86, 95% CI = 2.15 to 9.57) and delayed initiation (SMD = 2.80, 95% CI = 0.79 to 4.81) were associated with significantly improved BBB scores. Similarly, significant effects were observed for short-course treatment (≤3 days; SMD = 4.63, 95% CI = 2.58 to 6.68) and long-course treatment (≥7 days; SMD = 5.57, 95% CI = 0.91 to 10.22).

### 3.5 BMS and subgroup analysis

**Figure 4:**
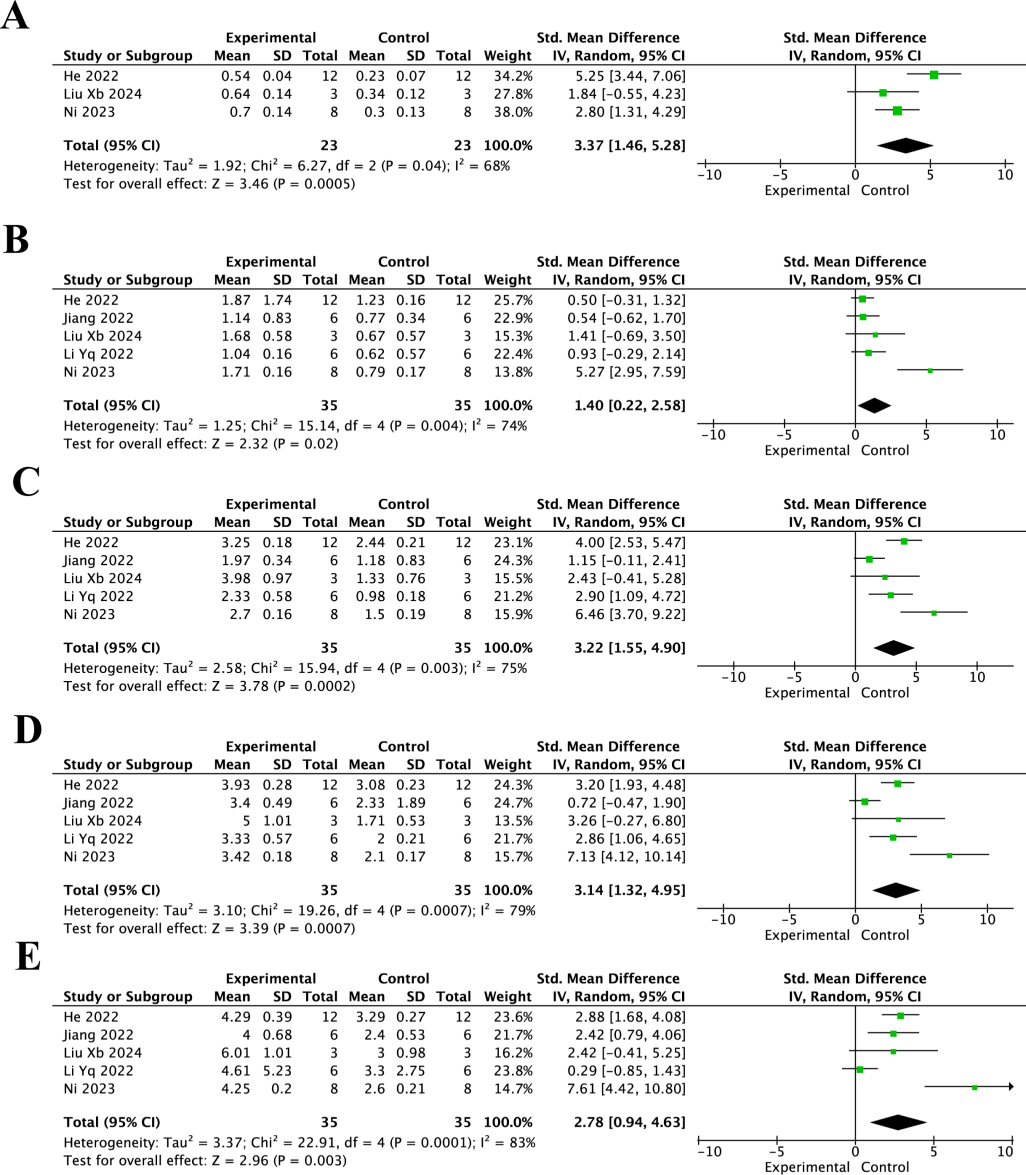
Forest plots of BMS scores for the effect of resveratrol on locomotor recovery after spinal cord injury at different post-injury time points. **(A)** 3 days after injury; **(B)** 7 days after injury; **(C)** 14 days after injury; **(D)** 21 days after injury; and **(E)** 28 days after injury.

As shown in **Figures 4A–E**, BMS scores in the resveratrol treatment group were significantly higher than those in the control group at different post-injury time points. At 3 days after injury (**Figure 4A**), the pooled analysis showed a significant increase in BMS scores in the resveratrol group compared with the control group (SMD = 3.37, 95% CI = 1.46 to 5.28, p < 0.001). At 7 days (**Figure 4B**), BMS scores remained significantly improved in the resveratrol group (SMD = 1.40, 95% CI = 0.22 to 2.58, p = 0.02). At 14 days (**Figure 4C**), the pooled effect size was SMD = 3.22 (95% CI = 1.55 to 4.90, p < 0.001). At 21 days (**Figure 4D**), resveratrol also significantly increased BMS scores compared with the control group (SMD = 3.14, 95% CI = 1.32 to 4.95, p < 0.001). At 28 days (**Figure 4E**), the beneficial effect remained significant (SMD = 2.78, 95% CI = 0.94 to 4.63, p < 0.001).

Further subgroup analyses of BMS scores were performed according to route of administration, dose, and treatment duration at different post-injury time points (**Supplementary Table 4**). At 7 days after SCI, a significant improvement in BMS score was observed in the intravenous injection subgroup (SMD = 0.82, 95% CI = 0.04 to 1.60, p = 0.04), whereas the non-intravenous injection subgroup did not reach statistical significance (SMD = 2.76, 95% CI = -1.91 to 7.42, p = 0.25). Similarly, the 5 mg/kg subgroup showed a significant effect (SMD = 0.82, 95% CI = 0.04 to 1.60, p = 0.04), while the 200 mg/kg subgroup did not (SMD = 2.76, 95% CI = -1.91 to 7.42, p = 0.25). Neither the ≤7-day treatment subgroup (SMD = 2.40, 95% CI = -0.07 to 4.87, p = 0.06) nor the >7-day treatment subgroup (SMD = 0.51, 95% CI = -0.15 to 1.18, p = 0.13) showed a statistically significant difference. At 14 days, significant improvements in BMS scores were detected in both the intravenous injection subgroup (SMD = 1.91, 95% CI = 0.72 to 3.10, p < 0.01) and the non-intravenous injection subgroup (SMD = 4.94, 95% CI = 2.60 to 7.29, p < 0.01). Consistently, both the 5 mg/kg subgroup (SMD = 1.91, 95% CI = 0.72 to 3.10, p < 0.01) and the 200 mg/kg subgroup (SMD = 4.94, 95% CI = 2.60 to 7.29, p < 0.01) showed significant effects. Significant improvements were also observed in both the ≤7-day treatment subgroup (SMD = 4.46, 95% CI = 0.52 to 8.41, p < 0.01) and the >7-day treatment subgroup (SMD = 2.64, 95% CI = 0.85 to 4.44, p < 0.01). At 21 days, both the intravenous injection subgroup (SMD = 1.93, 95% CI = 0.19 to 3.68, p = 0.03) and the non-intravenous injection subgroup (SMD = 4.92, 95% CI = 1.10 to 8.74, p = 0.01) showed significant increases in BMS scores. Likewise, significant effects were observed in the 5 mg/kg subgroup (SMD = 1.93, 95% CI = 0.19 to 3.68) and the 200 mg/kg subgroup (SMD = 4.92, 95% CI = 1.10 to 8.74). For treatment duration, the ≤7-day treatment subgroup remained significant (SMD = 4.30, 95% CI = 1.61 to 6.99, p < 0.01), whereas the >7-day treatment subgroup was not statistically significant (SMD = 1.95, 95% CI = -0.49 to 4.39, p = 0.12). At 28 days, the intravenous injection subgroup did not show a significant difference (SMD = 1.49, 95% CI = -0.14 to 3.13, p = 0.07), whereas the non-intravenous injection subgroup remained significant (SMD = 5.00, 95% CI = 0.39 to 9.61, p = 0.03). The same pattern was observed for dose, with no significant effect in the 5 mg/kg subgroup (SMD = 1.49, 95% CI = -0.14 to 3.13, p = 0.07) but a significant improvement in the 200 mg/kg subgroup (SMD = 5.00, 95% CI = 0.39 to 9.61, p = 0.03). In contrast, both treatment-duration subgroups showed significant effects, including the ≤7-day treatment subgroup (SMD = 1.67, 95% CI = 1.47 to 1.87, p < 0.01) and the >7-day treatment subgroup (SMD = 1.08, 95% CI = 0.83 to 1.33, p < 0.01).

### 3.6 MDA, SOD, GSH

**Figure 5:**
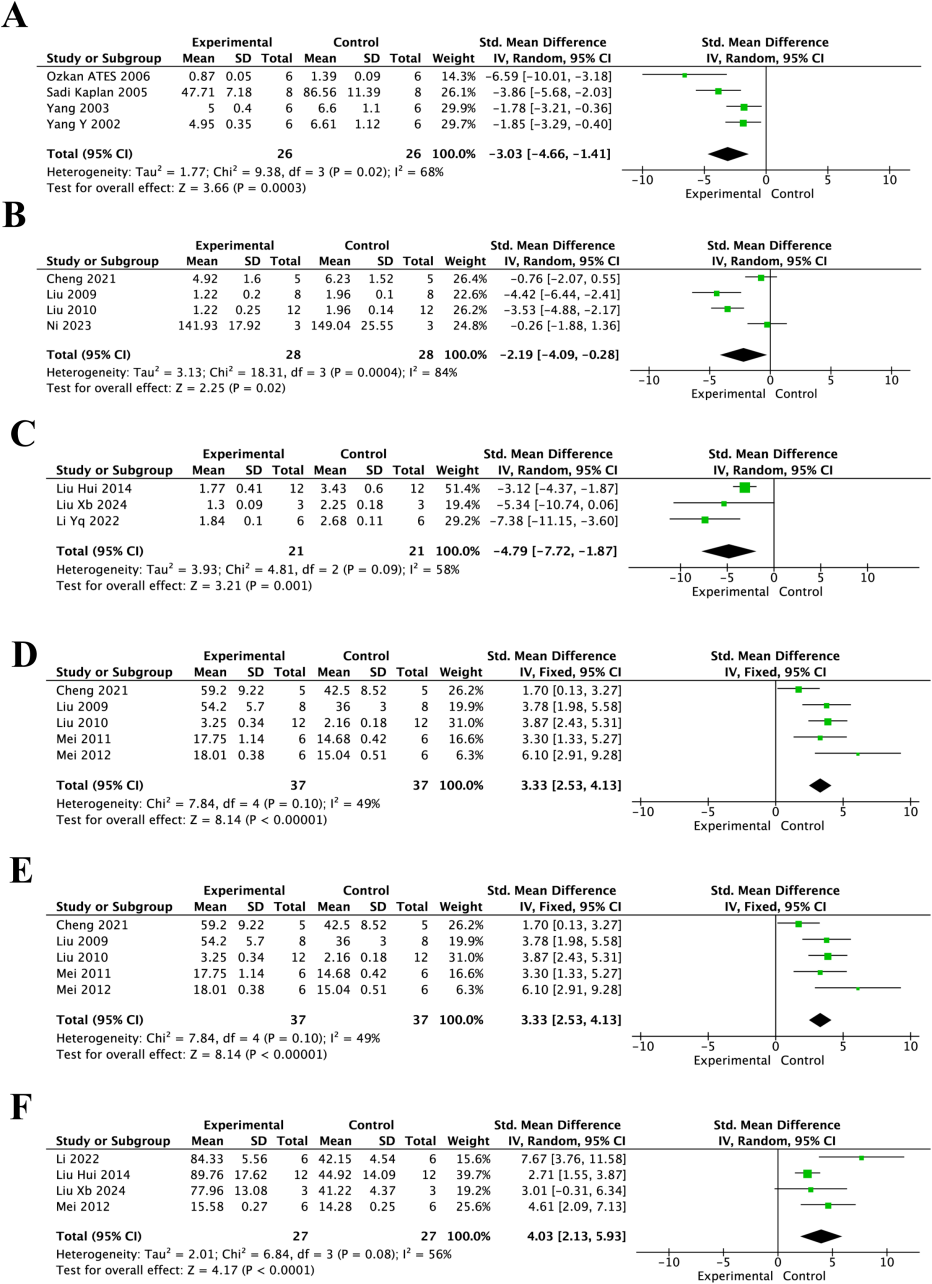
Forest plots of MDA levels and SOD activity for the effect of resveratrol on oxidative stress after spinal cord injury at different post-injury time points. **(A)** MDA levels at 24 h after injury; **(B)** MDA levels at 3 days after injury; **(C)** MDA levels at 7 days after injury; **(D)** SOD activity at 24 h after injury; **(E)** SOD activity at 3 days after injury; and **(F)** SOD activity at 7 days after injury.

**Figure 6:**
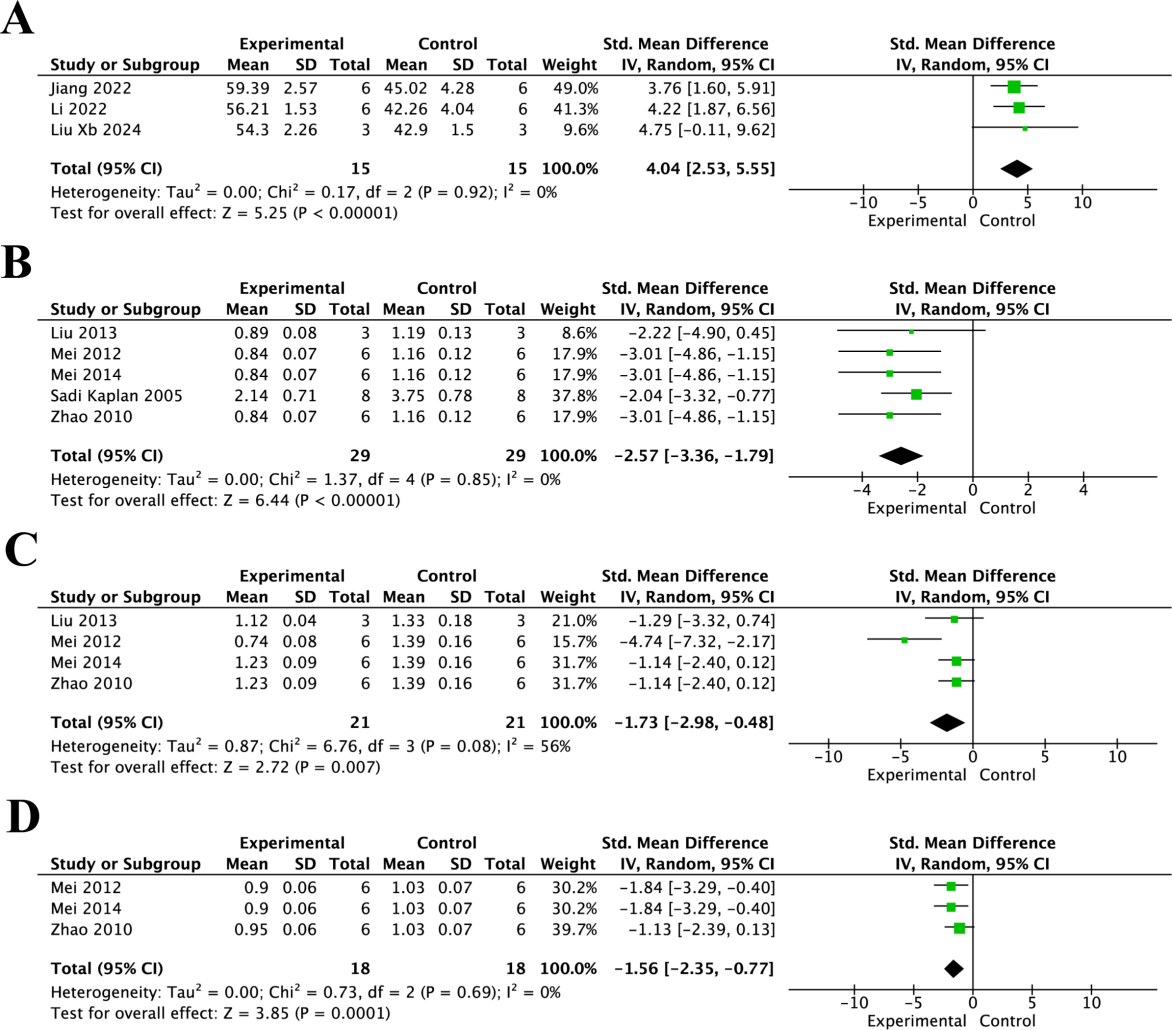
Forest plots of GSH and MPO levels for the effect of resveratrol on oxidative stress and inflammation after spinal cord injury at different post-injury time points. **(A)** GSH levels at 7 days after injury; **(B)** MPO levels at 24 h after injury; **(C)** MPO levels at 3 days after injury; and **(D)** MPO levels at 7 days after injury.

As shown in **Figures 5A–F**, resveratrol significantly modulated oxidative stress-related markers after spinal cord injury. For MDA, the pooled analysis showed that levels in the resveratrol group were significantly lower than those in the control group at 24 h (**Figure 5A**) (SMD = -3.03, 95% CI = -4.66 to -1.41, p < 0.01), 3 days (**Figure 5B**) (SMD = -2.19, 95% CI = -4.09 to -0.28, p = 0.02), and 7 days (**Figure 5C**) (SMD = -4.79, 95% CI = -7.72 to -1.87, p < 0.01). For SOD, pooled analysis demonstrated that activity in the resveratrol group was significantly higher than that in the control group at 24 h (**Figure 5D**) (SMD = 2.71, 95% CI = 1.90 to 3.51, p < 0.01), 3 days (**Figure 5E**) (SMD = 3.33, 95% CI = 2.53 to 4.13, p < 0.01), and 7 days (**Figure 5F**) (SMD = 4.03, 95% CI = 2.13 to 5.93, p < 0.01). As shown in **Figure 6A**, GSH levels in the resveratrol group were significantly higher than those in the control group at 7 days after injury (SMD = 4.04, 95% CI = 2.53 to 5.55, p < 0.01).

### 3.7 MPO, TNF-α, IL-1β, IL-6 and IL-10

**Figure 7:**
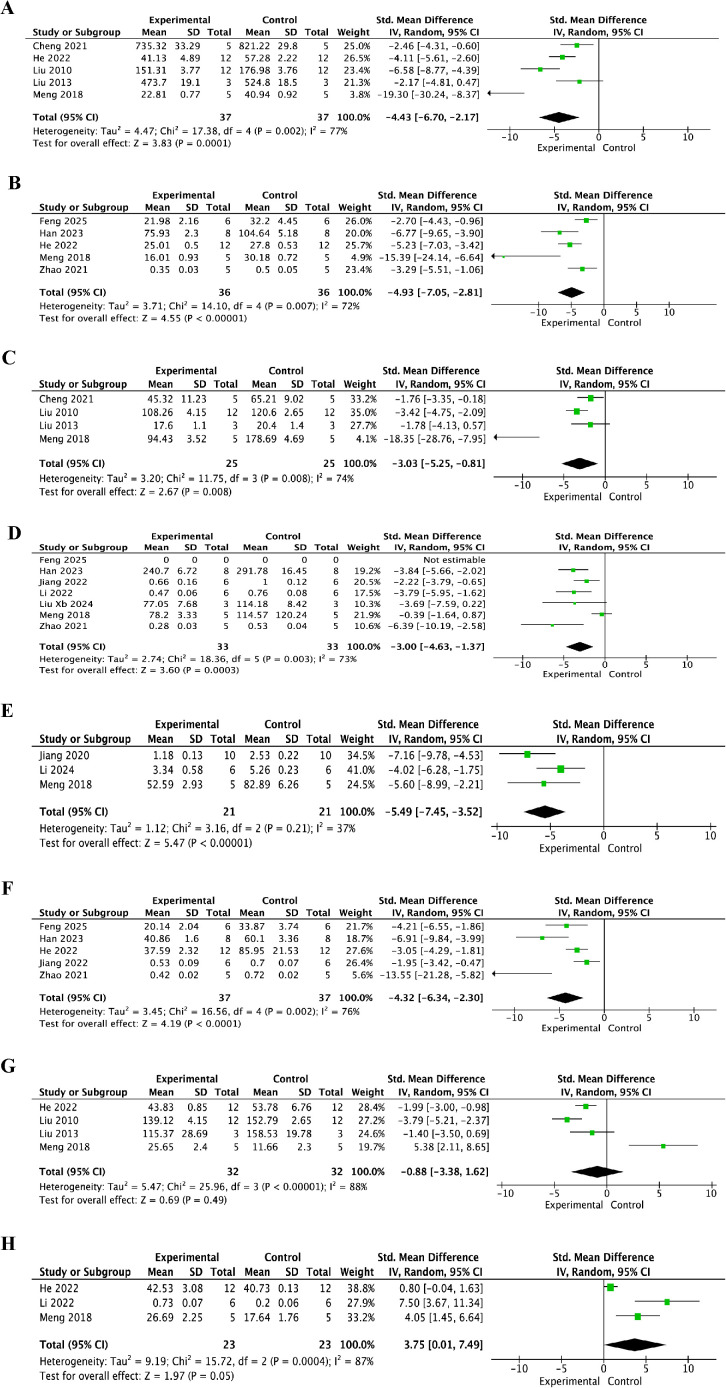
Forest plots of TNF-α, IL-1β, IL-6, and IL-10 levels for the effect of resveratrol on inflammatory responses after spinal cord injury at different post-injury time points. **(A)** TNF-α levels at 3 days after injury; **(B)** TNF-α levels at 7 days after injury; **(C)** IL-1β levels at 3 days after injury; **(D)** IL-1β levels at 7 days after injury; **(E)** IL-1β levels at 14 days after injury; **(F)** IL-6 levels at 7 days after injury; **(G)** IL-10 levels at 3 days after injury; and **(H)** IL-10 levels at 7 days after injury.

As shown in **Figures 6B–D**, MPO levels in the resveratrol group were significantly lower than those in the control group at 24 h (**Figure 6B**) (SMD = -2.57, 95% CI = -3.36 to -1.79, p < 0.01), 3 days (**Figure 6C**) (SMD = -1.73, 95% CI = -2.98 to -0.48, p < 0.01), and 7 days (**Figure 6D**) (SMD = -1.56, 95% CI = -2.35 to -0.77, p < 0.01). As shown in **Figures 7A, B**, TNF-α levels in the resveratrol group were significantly lower than those in the control group at 3 days (**Figure 7A**) (SMD = -4.43, 95% CI = -6.70 to -2.17, p < 0.001) and 7 days after injury (**Figure 7B**) (SMD = -4.93, 95% CI = -7.05 to -2.81, p < 0.001). As shown in **Figures 7C–E**, IL-1β levels in the resveratrol group were also significantly lower than those in the control group at 3 days (**Figure 7C**) (SMD = -3.03, 95% CI = -5.25 to -0.81, p < 0.001), 7 days (**Figure 7D**) (SMD = -3.00, 95% CI = -4.63 to -1.37, p < 0.001), and 14 days after injury (**Figure 7E**) (SMD = -5.49, 95% CI = -7.45 to -3.52, p < 0.001). As shown in **Figure 7F**, IL-6 levels in the resveratrol group were significantly lower than those in the control group at 7 days after injury (SMD = -4.32, 95% CI = -6.34 to -2.30, p < 0.01), indicating that resveratrol significantly suppressed IL-6 expression. As shown in **Figures 7G, H**, there was no significant difference in IL-10 levels between the resveratrol and control groups at 3 days after injury (**Figure 7G**) (SMD = -0.88, 95% CI = -3.38 to 1.62, p = 0.49). However, at 7 days after injury (**Figure 7H**), IL-10 showed a borderline increase in the resveratrol group compared with the control group (SMD = 3.75, 95% CI = 0.01 to 7.49, p = 0.05), which may indicate a possible enhancement of the anti-inflammatory response at the later stage after SCI.

### 3.8 TUNEL, BCL-2, BAX and Caspase-3

**Figure 8:**
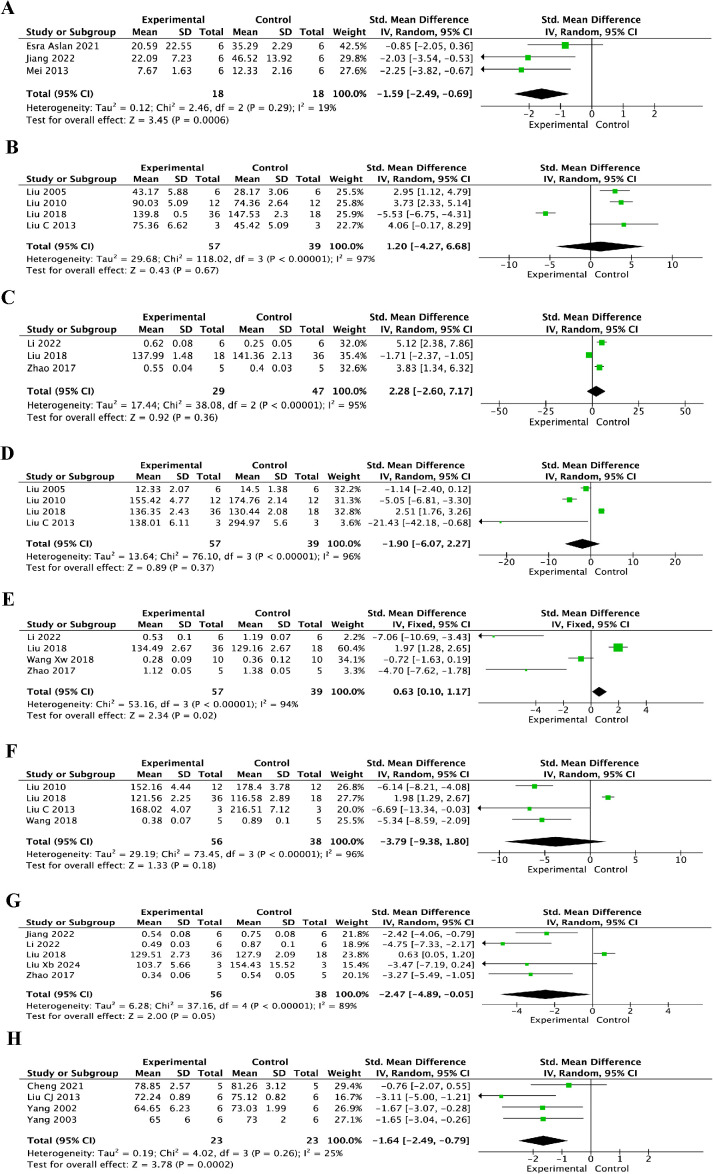
Forest plots of TUNEL-positive rate, BCL-2, BAX, caspase-3, and spinal cord water content for the effect of resveratrol on apoptosis and spinal cord edema after spinal cord injury at different post-injury time points. **(A)** TUNEL-positive rate at 7 days after injury; **(B)** BCL-2 levels at 3 days after injury; **(C)** BCL-2 levels at 7 days after injury; **(D)** BAX levels at 3 days after injury; **(E)** BAX levels at 7 days after injury; **(F)** caspase-3 levels at 3 days after injury; **(G)** caspase-3 levels at 7 days after injury; and **(H)** spinal cord water content after injury.

As shown in **Figure 8A**, the pooled analysis showed that resveratrol significantly reduced the TUNEL-positive rate at 7 days after injury (SMD = -1.59, 95% CI = -2.49 to -0.69, p < 0.01), suggesting a possible reduction in apoptotic cell death after SCI. However, the molecular apoptosis-related markers showed less consistent results. As shown in **Figures 8B, C**, no significant difference in BCL-2 levels was observed between the resveratrol and control groups at either 3 days (**Figure 8B**) (SMD = 1.20, 95% CI = -4.27 to 6.68, p = 0.67) or 7 days after injury (**Figure 8C**) (SMD = 2.28, 95% CI = -2.60 to 7.17, p = 0.36). As shown in **Figures 8D, E**, no significant difference in BAX levels was found between the two groups at 3 days after injury (**Figure 8D**) (SMD = -1.90, 95% CI = -6.07 to 2.27, p = 0.37), whereas BAX levels were significantly higher in the resveratrol group at 7 days (**Figure 8E**) (SMD = 0.63, 95% CI = 0.10 to 1.17, p = 0.02). As shown in **Figures 8F, G**, no significant difference in caspase-3 levels was observed between the resveratrol and control groups at 3 days after injury (**Figure 8F**) (SMD = -3.79, 95% CI = -9.38 to 1.80, p = 0.18), while a borderline decrease was observed at 7 days after injury (**Figure 8G**) (SMD = -2.47, 95% CI = -4.89 to -0.05, p = 0.05). Taken together, these findings suggest that the strongest evidence for a possible anti-apoptotic effect of resveratrol comes from TUNEL-based outcomes, whereas the molecular evidence involving BCL-2, BAX, and caspase-3 remains heterogeneous.

### 3.9 Spinal cord edema

As shown in **Figure 8H**, spinal cord water content was significantly lower in the resveratrol group than in the control group (SMD = -1.64, 95% CI = -2.49 to -0.79, p < 0.001), suggesting a potential effect of resveratrol on reducing spinal cord edema after SCI. This interpretation was based on spinal cord water content as the available edema-related outcome.

### 3.10 Sensitivity analysis

As shown in **Supplementary Figure 1**, after leave-one-out sensitivity analyses of BBB and BMS scores at each observation time point, omission of any single study did not materially alter the pooled effect estimates, and the overall direction of effect remained unchanged. These findings indicate that the results were generally robust and reliable. Although some heterogeneity persisted at certain time points, the sensitivity analyses suggested that it was not primarily driven by any individual study, but was more likely attributable to differences across studies in animal model induction, dosing regimens, timing of intervention, treatment duration, and outcome assessment time points.

## 4 Discussion

This systematic review and meta-analysis demonstrated that resveratrol exerts significant overall protective effects in animal models of spinal cord injury. The findings showed that resveratrol not only consistently improved post-injury locomotor outcomes, but also intervened in several key pathological processes, including attenuation of oxidative stress, suppression of inflammation, modulation of apoptosis-related changes, and alleviation of spinal cord edema. Specifically, resveratrol significantly increased BBB and BMS scores, suggesting its potential to promote neurological functional recovery. At the same time, the reductions in MDA, MPO, TNF-α, IL-1β, and IL-6 levels, together with the increases in SOD, GSH, and late-stage IL-10 levels, indicate that resveratrol possesses marked antioxidant and anti-inflammatory activities. In addition, the decrease in TUNEL-positive cell rate suggests a possible reduction in apoptotic cell death, while the reduction in spinal cord water content indicates alleviation of tissue edema. Taken together, these findings suggest that resveratrol may improve overall neurological outcomes after SCI by modulating the secondary injury cascade through multiple targets and pathways.

In terms of locomotor recovery, the sustained improvement in BBB and BMS scores provides the most direct and overall convincing evidence in the present study (64, 65). Our results showed that resveratrol significantly increased BBB scores at 3, 7, 14, 21, and 28 days after injury, and similarly improved BMS scores at the corresponding time points, suggesting that its neurofunctional benefits were not restricted to the acute phase but extended throughout the early and subacute stages of recovery after SCI. This relatively consistent promotive effect on locomotor recovery is in line with previous meta-analytic findings. For example, a meta-analysis by Xu et al. (66), which included 12 rat studies, reported that resveratrol significantly improved BBB scores, with a pooled effect size of MD = 3.85 (95% CI 2.10 to 5.59, P < 0.01), indicating a clear benefit for hindlimb motor recovery after SCI. Notably, the time-dependent pattern observed in our study, in which the beneficial effect was maintained across multiple post-injury time points, is also similar to findings from other preclinical meta-analyses in SCI. For instance, Zhou et al. (67), in a systematic review and network meta-analysis of metformin, found that the weighted mean difference in BBB score increased from 0.42 (95% CI -0.01 to 0.85) on day 3 to 3.48 (95% CI = 2.04 to 4.92) on day 28, suggesting that behavioral benefits may accumulate progressively over the course of recovery.

Subgroup analyses suggested that the beneficial effects of resveratrol on locomotor recovery after SCI may be influenced by dose, timing of administration, and treatment duration. For BBB scores, the high-dose subgroup generally showed larger effect sizes than the low-dose subgroup at multiple time points, particularly after 14 days, suggesting a possible dose-dependent effect. Both immediate and delayed administration tended to improve locomotor recovery at most time points, although delayed administration did not reach statistical significance at 21 days, indicating that earlier intervention may be more favorable for long-term functional improvement; this finding is consistent with the pathological process of SCI, in which secondary injury is rapidly initiated after trauma and continues to evolve over time (68). Although different treatment regimens all showed overall positive effects, their advantageous time windows were not entirely consistent, suggesting that resveratrol may exert neuroprotective effects through modulation of both the acute injury response and the secondary injury cascade, which is also in line with previous reviews indicating that resveratrol promotes functional recovery after SCI through antioxidant, anti-inflammatory, and anti-apoptotic mechanisms (32). In contrast, the subgroup findings for BMS scores were more variable, and some subgroups defined by route of administration, dose, or treatment duration did not reach statistical significance. Given that subgroup analyses generally require a larger number of studies to achieve sufficient statistical power, and considering the differences among included studies in injury models, treatment protocols, and observation time points, this inconsistency is more likely to reflect limited study numbers and between-study heterogeneity rather than the absence of a true therapeutic effect (69). Overall, the available evidence consistently supports a beneficial effect of resveratrol on locomotor recovery after SCI, although the optimal dose, route of administration, and treatment duration still require further clarification in well-designed animal studies.

Oxidative stress is one of the major drivers of secondary injury after SCI, and our findings indicate that resveratrol exerts substantial effects on this pathological process (70). The present meta-analysis showed that resveratrol significantly reduced MDA levels at 24 h, 3 days, and 7 days after injury, while significantly increasing SOD levels within a similar time window and elevating GSH content at 7 days. These findings are generally consistent with previous meta-analytic evidence from SCI animal studies. Xu et al. (66) reported that resveratrol significantly increased SOD levels (SMD = 5.22, 95% CI 2.98 to 7.45, P < 0.01) and significantly decreased MDA levels (SMD = -3.64, 95% CI -5.84 to -1.43, P = 0.001). Because MDA is a classic end product of lipid peroxidation, whereas SOD and GSH represent key enzymatic and non-enzymatic components of antioxidant defense, the reduction in MDA together with the increases in SOD and GSH suggests that resveratrol may not only attenuate oxidative damage but also enhance endogenous antioxidant capacity (70). The consistent directional changes in MDA, SOD, and GSH provide good internal coherence, thereby strengthening the robustness of the evidence for the antioxidant effects of resveratrol. Notably, these effects persisted from 24 h to 7 days after injury, suggesting that the action of resveratrol is not limited to transient free radical scavenging during the acute phase, but may also involve sustained regulation of redox homeostasis to mitigate secondary tissue damage (71). Given the close association of oxidative stress with mitochondrial dysfunction, membrane disruption, and neuronal death, these findings also provide an important mechanistic basis for the beneficial effects of resveratrol on subsequent neurological recovery.

MPO levels were significantly reduced at 24 h, 3 days, and 7 days, suggesting that resveratrol may suppress early inflammatory cell infiltration or decrease the activity of inflammation-related enzymes (72)​ At the same time, tumor necrosis factor-α (TNF-α) was significantly decreased at 3 and 7 days, interleukin-1β (IL-1β) at 3, 7, and 14 days, and interleukin-6 (IL-6) at 7 days, which is consistent with previous findings that resveratrol can inhibit the expression of pro-inflammatory cytokines (27). This anti-inflammatory effect is also supported by studies showing that resveratrol can suppress activation of the nuclear factor-κB (NF-κB) signaling pathway (73)​. Overall, these findings indicate that resveratrol alleviates inflammation after SCI primarily by downregulating pro-inflammatory mediators. Notably, IL-10 showed no significant difference at 3 days but showed a borderline increase at 7 days in the present study. Previous studies have shown that, as an important anti-inflammatory cytokine, IL-10 generally increases later than acute-phase pro-inflammatory mediators and is mainly involved in inflammation resolution and tissue protection (74, 75). Therefore, the anti-inflammatory effects of resveratrol may not only depend on suppression of pro-inflammatory factors, but may also involve a possible reshaping of the post-injury inflammatory microenvironment.

Current evidence suggests that resveratrol may attenuate apoptosis after spinal cord injury, but this interpretation should be made with caution because apoptosis-related outcomes were not fully consistent across indicators. The strongest evidence comes from the reduction in TUNEL-positive cells, which reflects the terminal outcome of apoptotic cell death. Previous studies have also suggested that resveratrol can reduce DNA damage and cell apoptosis and exert protective effects on neurons (76). However, the findings for other apoptosis-related molecular markers were less uniform. In the present meta-analysis, no significant changes were observed in BCL-2 at 3 and 7 days or in caspase-3 at 3 days, while BAX was unexpectedly increased at 7 days, which does not fully conform to the classical anti-apoptotic pattern. One possible explanation is that TUNEL mainly reflects the terminal outcome of apoptosis, whereas BCL-2, BAX, and caspase-3 represent specific molecular nodes within apoptotic pathways and are therefore more susceptible to factors such as sampling time, detection method, injury site, and quantification criteria. In addition, the limited number of available studies may have reduced the stability of the pooled estimates. Therefore, the anti-apoptotic interpretation should be regarded as mainly supported by the reduction in TUNEL-positive cells, whereas molecular evidence remains exploratory and requires further validation. Consistent with previous reports, the neuroprotective effects of resveratrol are unlikely to depend on a single apoptotic pathway alone, but may instead arise from the combined actions of antioxidative, anti-inflammatory, and autophagy-regulating mechanisms. For example, resveratrol has been reported to activate autophagy and suppress apoptosis through signaling pathways such as SIRT1/AMPK, thereby attenuating secondary injury after SCI (26).

Of course, the findings of this study should still be interpreted with caution. First, although the overall direction of effect was generally consistent, some subgroup analyses and apoptosis-related molecular outcomes showed instability or even inconsistency, suggesting that the current evidence may still be influenced by small-study effects and between-study heterogeneity. Second, substantial differences across studies in injury models, dosage, timing of treatment initiation, route of administration, and treatment duration may have affected the precision and comparability of the pooled estimates. Third, only a limited number of studies were available for some outcomes, resulting in low statistical power and potentially greater fluctuations in effect size estimates. Finally, the extent to which findings from animal experiments can be directly translated into clinical practice remains uncertain, and the optimal dose, pharmacokinetic profile, and long-term safety of resveratrol in human SCI still require further investigation. Therefore, future studies should adopt more standardized experimental designs, provide more complete reporting, and conduct deeper mechanistic validation, while gradually advancing translational research to enhance the clinical relevance of the available evidence.

## 5 Conclusion

In conclusion, the present study demonstrates that resveratrol exerts clear neuroprotective effects in animal models of spinal cord injury. The meta-analysis showed that resveratrol significantly improved BBB and BMS scores, indicating sustained promotion of locomotor recovery after injury. At the same time, the reductions in MDA, MPO, TNF-α, IL-1β, and IL-6 levels, together with the increases in SOD and GSH and the borderline increase in late-stage IL-10, suggest that resveratrol may alleviate oxidative stress and modulate the inflammatory microenvironment at multiple stages after SCI. In addition, the decrease in TUNEL-positive rate suggests a possible reduction in apoptotic cell death, while the reduction in spinal cord water content suggests a potential effect of resveratrol on spinal cord edema, although this conclusion was based on spinal cord water content as the available edema-related outcome. Overall, resveratrol may improve neurological outcomes after SCI by simultaneously targeting several key components of secondary injury, including oxidative stress, inflammation, apoptosis-related changes, and tissue edema. These findings support the potential value of resveratrol as a multitarget candidate therapy for SCI and provide an experimental basis for future mechanistic studies, optimization of treatment regimens, and translational research.

## Data Availability

The original contributions presented in the study are included in the article/**Supplementary Material**. Further inquiries can be directed to the corresponding author.

## Supplementary material

Supplementary Figure 1 Leave-one-out sensitivity analyses for pooled BBB and BMS scores at different post-injury time points. **(A)** BBB score at 3 days; **(B)** BBB score at 7 days; **(C)** BBB score at 14 days; **(D)** BBB score at 21 days; **(E)** BBB score at 28 days; **(F)** BMS score at 3 days; **(G)** BMS score at 7 days; **(H)** BMS score at 14 days; **(I)** BMS score at 21 days; and **(J)** BMS score at 28 days.

Supplementary Table 1 Subgroup analysis of BBB score at 3 days after SCI following resveratrol treatment.

Supplementary Table 2 Subgroup analyses of BBB scores at 7, 14, and 21 days after SCI following resveratrol treatment.

Supplementary Table 3 Subgroup analysis of BBB score at 28 days after SCI following resveratrol treatment.

Supplementary Table 4 Subgroup analyses of BMS scores at 7, 14, 21, and 28 days after SCI following resveratrol treatment.
