# Supplementary figures and images for: Protective effect and possible mechanisms of resveratrol in animal models of spinal cord injury: a preclinical systematic review and meta-analysis
Source: Front Immunol. 2026 May 21;17:1853441. doi: 10.3389/fimmu.2026.1853441 (PMC13233272; doi:10.3389/fimmu.2026.1853441)

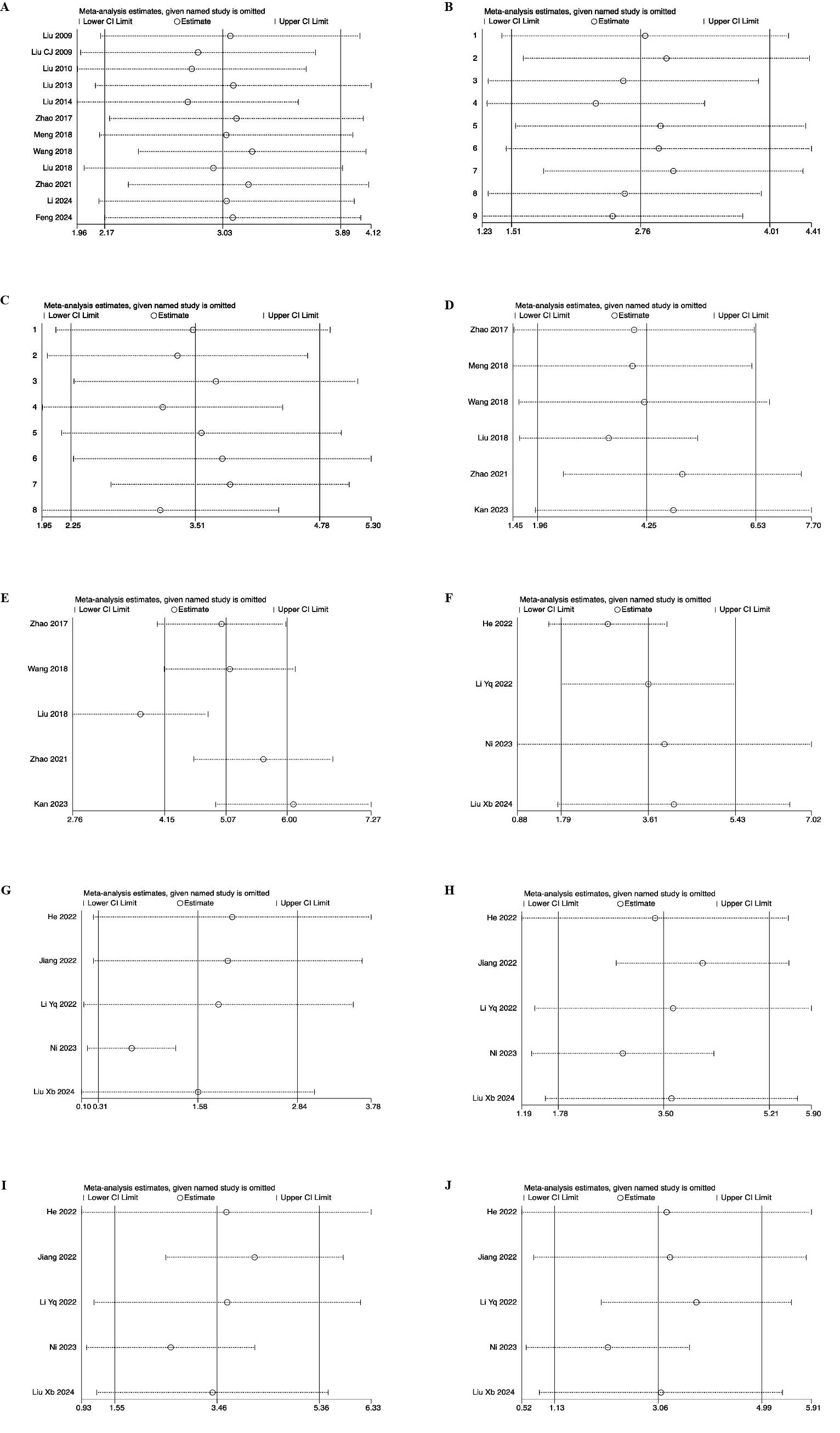

Supplement: Supplementary Figure 1 — Leave-one-out sensitivity analyses for pooled BBB and BMS scores at different post-injury time points. (A) BBB score at 3 days; (B) BBB score at 7 days; (C) BBB score at 14 days; (D) BBB score at 21 days; (E) BBB score at 28 days; (F) BMS score at 3 days; (G) BMS score at 7 days; (H) BMS score at 14 days; (I) BMS score at 21 days; and (J) BMS score at 28 days. [file Image1.jpeg]
