# Supplementary material for: Protective effect and possible mechanisms of resveratrol in animal models of spinal cord injury: a preclinical systematic review and meta-analysis
Source: Front Immunol. 2026 May 21;17:1853441. doi: 10.3389/fimmu.2026.1853441 (PMC13233272; doi:10.3389/fimmu.2026.1853441)
Supplement: Supplementary Table 1 — Subgroup analysis of BBB score at 3 days after SCI following resveratrol treatment. [file SupplementaryFile1.docx]

| Supplementary Table 1. Subgroup analysis of BBB score at 3 days after SCI following resveratrol treatment | | | |
| --- | --- | --- | --- |
| **Subgroup** | **SMD (95% CI)** | **I²** | **p value** |
| **Dose category** |  |  |  |
| ≤50 mg/kg | 1.69 [0.19, 3.18] | 58 | < 0.01 |
| 100 mg/kg | 2.76 [1.98, 3.54] | 30 | < 0.01 |
| ≥200 mg/kg | 3.85 [1.88, 5.81] | 88 | < 0.01 |
| **Timing of treatment initiation** |  |  |  |
| Immediate initiation | 2.85 [1.93, 3.78] | 74 | < 0.01 |
| Delayed initiation | 2.91 [0.39, 5.44] | 86 | < 0.01 |
| **Treatment regimen** |  |  |  |
| Single-dose administration | 2.82 [1.58, 4.06] | 50 | < 0.01 |
| 3-day administration | 3.54 [1.35, 5.73] | 89 | < 0.01 |
| ≥7-day administration | 2.40 [1.09, 3.72] | 74 | < 0.01 |

| Supplementary Table 2. Subgroup analyses of BBB scores at 7, 14, and 21 days after SCI following resveratrol treatment | | | | | | | | | |
| --- | --- | --- | --- | --- | --- | --- | --- | --- | --- |
|  | **7 days, SMD (95% CI)** | **I²** | **p value** | **14 days, SMD (95% CI)** | **I²** | **p value** | **21 days, SMD (95% CI)** | **I²** | **p value** |
| **Dose category** |  |  |  |  |  |  |  |  |  |
| <100 mg/kg | 1.11 [0.33, 1.88] | 31 | <0.01 | 1.88 [1.01, 2.75] | 26 | <0.01 | 1.42 [0.46, 2.39] | 19 | <0.01 |
| ≥100 mg/kg | 3.42 [1.85, 4.99] | 78 | <0.01 | 4.14 [2.71, 5.57] | 57 | <0.01 | 5.93 [4.72, 7.15] | 9 | <0.01 |
| **Timing of treatment initiation** |  |  |  |  |  |  |  |  |  |
| Immediate initiation | 2.82 [0.99, 4.66] | 89 | <0.01 | 3.16 [1.45, 4.88] | 83 | <0.01 | 4.65 [2.19, 7.12] | 86 | <0.01 |
| Delayed initiation | 2.01 [0.82, 3.20] | 48 | <0.01 | 3.51 [1.29, 5.73] | 70 | <0.01 | 0.87 [-0.46, 2.20] | — | 0.2 |
| **Treatment regimen** |  |  |  |  |  |  |  |  |  |
| Single-dose administration | 1.56 [0.81, 2.31] | 0 | <0.01 | 3.00 [1.57, 4.43] | 36 | <0.01 | 5.39 [3.07, 7.71] | 0 | <0.01 |
| Repeated administration | 3.16 [1.26, 5.06] | 89 | <0.01 | 3.34 [1.49, 5.19] | 85 | <0.01 | 3.38 [0.60, 6.17] | 93 | <0.01 |

| Supplementary Table 3. Subgroup analysis of BBB score at 28 days after SCI following resveratrol treatment | | | |
| --- | --- | --- | --- |
| **Subgroup** | **SMD (95% CI)** | **I²** | **p value** |
| **Dose category** |  |  |  |
| <100 mg/kg | 3.09 [1.88, 4.30] | 0 | <0.01 |
| ≥100 mg/kg | 6.81 [2.42, 11.20] | 88 | <0.01 |
| **Timing of treatment initiation** |  |  |  |
| Immediate initiation | 5.86 [2.15, 9.57] | 91 | <0.01 |
| Delayed initiation | 2.80 [0.79, 4.81] | — | <0.01 |
| **Treatment regimen** |  |  |  |
| Short-course administration (≤3 days) | 4.63 [2.58, 6.68] | 0 | <0.01 |
| Long-course administration (≥7 days) | 5.57 [0.91, 10.22] | 95 | 0.02 |

| Supplementary Table 4. Subgroup analyses of BMS scores at 7, 14, 21, and 28 days after SCI following resveratrol treatment | | | | | | | | | | | | |
| --- | --- | --- | --- | --- | --- | --- | --- | --- | --- | --- | --- | --- |
| **Subgroup** | **7 days, SMD (95% CI)** | **I²** | **p value** | **14 days, SMD (95% CI)** | **I²** | **p value** | **21 days, SMD (95% CI)** | **I²** | **p value** | **28 days, SMD (95% CI)** | **I²** | **p value** |
| **Route of administration** |  |  |  |  |  |  |  |  |  |  |  |  |
| Intravenous injection | 0.82 [0.04, 1.60] | 0 | 0.04 | 1.91 [0.72, 3.10] | 24 | <0.01 | 1.93 [0.19, 3.68] | 59 | 0.03 | 1.49 [-0.14, 3.13] | 62 | 0.07 |
| Non-intravenous injection | 2.76 [-1.91, 7.42] | 93 | 0.25 | 4.94 [2.60, 7.29] | 58 | <0.01 | 4.92 [1.10, 8.74] | 82 | 0.01 | 5.00 [0.39, 9.61] | 86 | 0.03 |
| **Dose category** |  |  |  |  |  |  |  |  |  |  |  |  |
| 5 mg/kg | 0.82 [0.04, 1.60] | 0 | 0.04 | 1.91 [0.72, 3.10] | 24 | <0.01 | 1.93 [0.19, 3.68] | 82 | 0.01 | 1.49 [-0.14, 3.13] | 62 | 0.07 |
| 200 mg/kg | 2.76 [-1.91, 7.42] | 93 | 0.25 | 4.94 [2.60, 7.29] | 58 | <0.01 | 4.92 [1.10, 8.74] | 59 | 0.03 | 5.00 [0.39, 9.61] | 86 | 0.03 |
| **Treatment duration** |  |  |  |  |  |  |  |  |  |  |  |  |
| ≤7 days | 2.40 [-0.07, 4.87] | 81 | 0.06 | 4.46 [0.52, 8.41] | 75 | <0.01 | 4.30 [1.61, 6.99] | 66 | <0.01 | 1.67 [1.47, 1.87] | 28 | <0.01 |
| >7 days | 0.51 [-0.15, 1.18] | 0 | 0.13 | 2.64 [0.85, 4.44] | 77 | <0.01 | 1.95 [-0.49, 4.39] | 87 | 0.12 | 1.08 [0.83, 1.33] | 60 | <0.01 |
